# Span Efficiency of Flying Animals

**DOI:** 10.1093/icb/icag077

**Published:** 2026-06-10

**Authors:** Hiroki Yasuda, Madeleine R Inglis, Yosuke Yamamoto, Per Henningsson, Toshiyuki Nakata, Richard J Bomphrey

**Affiliations:** Graduate School of Science and Engineering, Chiba University, 1-33, Yayoi-cho, Inage-ku, Chiba-shi, Chiba 263-8522, Japan; Scottish Centre for Ecology and the Natural Environment, School of Biodiversity, One Health, and Veterinary Medicine, College of Medical, Veterinary and Life Sciences, University of Glasgow, Glasgow G63 0AW, UK; Graduate School of Science and Engineering, Chiba University, 1-33, Yayoi-cho, Inage-ku, Chiba-shi, Chiba 263-8522, Japan; Danish Hydraulic Institute (DHI), Agern Allé 5, DK-2970 Hørsholm, Denmark; Graduate School of Engineering, Chiba University, 1-33, Yayoi-cho, Inage-ku, Chiba-shi, Chiba 263-8522, Japan; Structure and Motion Laboratory, Royal Veterinary College, AL9 7TA Hatfield, UK

## Abstract

Span efficiency, a metric representing the efficiency of lift generation in flying animals, has been measured for birds, bats, and insects using particle image velocimetry over the past two decades. However, inconsistencies in capture and data analysis methods have introduced systematic biases, preventing a unified and fair comparison across diverse taxonomic groups and body sizes. Here, we quantify the impact of these methodological discrepancies using a computational fluid dynamics method, then standardize and synthesise span efficiency data from all thirty species measured to date. We apply a phylogenetic comparative method to evaluate how morphological and kinematic parameters affect span efficiency. Our analysis reveals that efficiency strongly correlates with body mass, suggests that larger flying animals may face stronger selective pressure for high span efficiency. Furthermore, simple theoretical span efficiency predictions for gliding and rotating flight—derived from wing planform and theoretical models—are correlated empirical values enabling reliable predictions of span efficiency in species that have not been measured. As an example of this morphology-based theoretical prediction, we compare the orange oakleaf butterfly, which has a wing planform that closely resembles a leaf for cryptic camouflage, with the migratory monarch butterfly. The monarch butterfly has a substantially higher predicted efficiency, quantifying the aerodynamic cost associated with mimicry. Ultimately, this study sheds new light on the interplay between physical constraints and lineage-specific adaptive strategies in biological flight, while providing a versatile framework to evaluate an aspect of flight performance from wing morphology without the need for intensive wind tunnel experiments.

## Introduction

Active flight is a pivotal locomotive adaptation that has evolved independently in insects, birds, bats and pterosaurs, driven by the immense ecological advantages it confers. This capability has allowed these lineages to avoid capture, access airborne or distinct food resources, and expand their habitats across vast geographical ranges through migration (Dudley 2002). Consequently, flight performance directly influences survival rates and reproductive success, acting as a primary target for natural selection across diverse taxa.

Among various determinants of flight capability, span efficiency (*e*_i_) serves as a critical aerodynamic metric that provides insights into ecologically-relevant energetic performance (Bomphrey 2006, 2012; Bomphrey et al. 2006; Muijreset al. 2012b; Henningsson and Bomphrey 2013). Defined as the ratio of the ideal minimum induced power to the actual induced power for a given lift and wing span, *e*_i_ quantifies how efficiently a flyer generates lift relative to the aerodynamic cost (Henningsson and Bomphrey 2012). A higher span efficiency, approaching the ideal of *e*_i_ = 1, implies a relative reduction in the power required to support body weight, thereby extending the distance an organism can travel on limited energy reserves, increasing the maximum load it can carry during foraging, or improve agility and acceleration due to higher potential force generation.

**Fig. 1:**
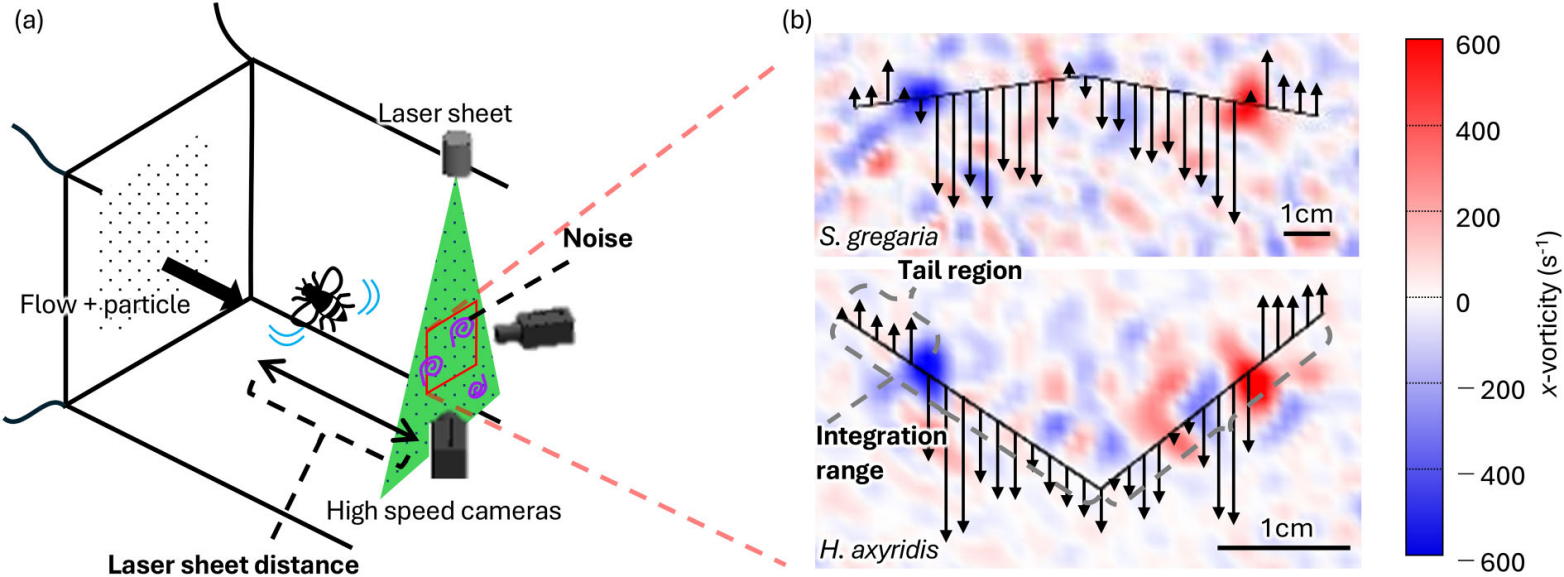
Example of flow velocity measurements for determining span efficiency. (A) Wake measurement of an insect in a wind tunnel using PIV. (B) Representative wakes of tethered insects (*Schistocerca gregaria* and *Harmonia axyridis*) in a wind tunnel. A transect is defined by the lines connecting the body centre to the wingtip vortices on both sides, and span efficiency is calculated from the velocity distribution extracted along this transect (including the “tails” extending the transect to cover the portion of the vortex with upwash). Vectors along the transects are subsampled for clarity.

To date, span efficiency has been experimentally quantified for 30 species, encompassing the three distinct lineages of powered flight: insects, birds, and bats (Muijres et al. 2011a, 2011b; Henningsson and Bomphrey 2012, 2013; Hubel et al. 2012; Johansson et al. 2012; Muijres et al. 2012a; Von Busse et al. 2012; Henningsson et al. 2014; Bomphrey et al. 2016; KleinHeerenbrink and Hedenström 2016; Song et al. 2022). These studies have been primarily based on particle image velocimetry (PIV) used in wind tunnel settings. In this method, the airflow is seeded with neutrally buoyant particles, illuminated by a laser sheet and imaged by high-speed cameras that detect the scattered light (Adrian 1991; Fig. 1A). By cross-corelating greyscale patterns within interrogation windows in consecutive images to determine the displacement of these particles over short time intervals, the flow velocity distribution can be resolved. While this technique fundamentally allows measurements in any user-defined region—such as streamwise-vertical planes to estimate time-dependent, unsteady aerodynamic forces (Spedding et al. 2003; Bomphrey et al. 2005; Stalnov et al. 2015)—the conventional framework for estimating span efficiency relies specifically on the flow velocity field within a vertical transverse plane located downstream of the animal, oriented perpendicular to the direction of flight. Transects through the computed velocity vector fields are then placed within the flow fields captured behind the animal to quantify the distribution of downwash velocity behind the trailing edge (Fig. 1B). In the context of flapping flight, span efficiency is typically evaluated as a cycle-averaged value. By integrating the momentum flux in the wake over a complete wingbeat, this approach inherently accounts for the net contribution of unsteady aerodynamic forces produced throughout the stroke cycle. By capturing the complex wake topography in this manner, literature reports have covered a broad spectrum of body sizes and wing morphologies, providing a foundational dataset for understanding this component of biological flight efficiency.

A critical challenge hindering the synthesis of these findings has been methodological inconsistency across studies; experimental conditions and data processing protocols have varied between research groups. For instance, calculated span efficiency can be significantly biased by the streamwise distance to the measurement plane due to wake roll-up and dissipation (Horstmann et al. 2014; Henningsson et al. 2015), the length of the transects used for integration, and PIV measurement uncertainties arising from cross-correlation errors or unsuitable experimental settings (Huang et al. 1997; Raffel et al. 2018). In some studies, the transects, representing the integration domain, have been extended beyond the wing tip vortex core axis to capture the upwash (referred to here as the “tails” of the transects; Fig. 1B), whereas in others they have not, which has introduced systematic biases. Since the induced flow field extends outboard of the wingtip vortex, truncating this integration domain at different points impacts the computed span efficiency. Similarly, if the streamwise distance at which the transect is taken varies, there could be error introduced owing to the wake structure being not static but evolving as it convects downstream. As the wake deforms behind the wings, Taylor’s frozen wake assumption is violated and measurements taken at different distances, at any given flight speed, may yield different span efficiency values (Horstmann et al. 2014; Henningsson et al. 2015). Furthermore, the fidelity of velocity fields derived from PIV measurements can vary between facilities due to differences in experimental constraints—such as imaging resolution, illumination intensity, and particle seeding density—which introduce varying levels of measurement uncertainty (Huang et al. 1997; Raffel et al. 2018). These heterogeneous measurement conditions have precluded a direct, unified comparison of flight efficiency across different animal sizes and lineages, leaving the overarching evolutionary trends of span efficiency largely unexplored and indeed unexplorable.

To address these methodological inconsistencies and develop a synthesis of existing data, we first established a correction framework based on computational fluid dynamics (CFD) to standardize the experimental data. Then, by integrating these corrected span efficiency values with morphological data, we applied a phylogenetic comparative method (PCM) (Symonds and Blomberg 2014) to investigate the evolutionary determinants of flight efficiency across insects, birds, and bats. In parallel, we calculated theoretical span efficiencies using distinct analytical models appropriate for each flight mode: classical lifting-line theory (LLT) for gliding wings (Anderson 2011) and actuator disk or blade element theory for rotary (flapping) wings (Stepniewski and Keys 1984). These rely only on wing planform and do not include the complexity of twist and other deformations seen *in vivo*. We compared these theoretical predictions with the experimentally obtained span efficiencies to evaluate the extent to which static morphology explains actual aerodynamic performance across diverse lineages.

## Methods

### Data

We conducted an exhaustive literature survey to collate experimental data on span efficiency derived from PIV or Particle Tracking Velocimetry (PTV). In cases where the original studies presented data across multiple conditions (e.g., flight speeds, individuals, or number of trials), we calculated the arithmetic mean to represent the species. In addition to the published data, we incorporated newly measured data for eight further insect species, specifically one from Coleoptera, four Hymenoptera, and three Diptera. The measurement protocols for these species followed the methodology described in (Henningsson and Bomphrey 2013). The final dataset comprised 30 species (22 insects, 5 birds, and 3 bats). For each entry, we compiled associated information regarding morphology, kinematics, and experimental conditions. The span efficiency values compiled in this study represent cycle-averaged metrics. While the specific analytical methodologies in the original literature varied, all values represent the time-averaged aerodynamic performance over a full wingbeat, thereby incorporating the net effects of unsteady force generation.

To calculate wing shape metrics, suitable planform information of the body and wings was required. These contour data were primarily extracted from the original publications or related literature dealing with the same species. In cases where such data were unavailable, we supplemented the dataset by collecting high-quality, publicly available images. Images were processed using ImageJ (Abramoff et al. 2004) for binarization and contour extraction. Based on these contours, we calculated morphological parameters such as taper ratio and area moments of inertia. Body size and flapping kinematic parameters (e.g., body mass, flapping frequency) were retrieved from the original sources. When specific values were missing, we filled the gaps using data from other literature concerning the same species.

**Fig. 2:**
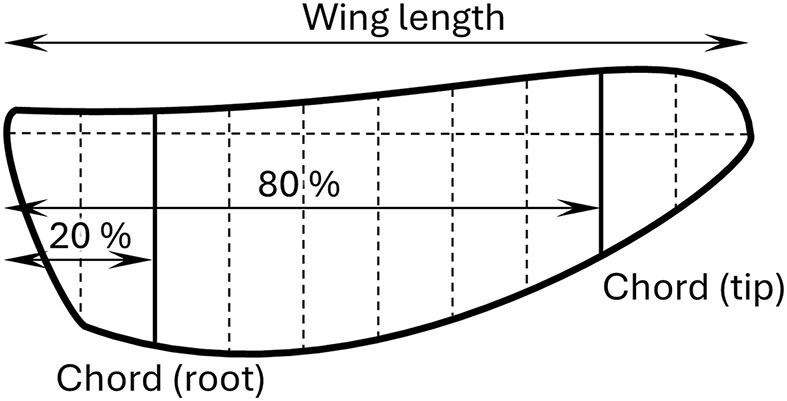
Definition of the taper ratio. The taper ratio is defined as the ratio between the chord lengths at 0.2*R* (*c*_root_) and 0.8*R* (*c*_tip_) of the wing length (*R*), measured from the wing root.

To facilitate comparative analysis across varying flight conditions and body sizes, we explicitly defined a set of dimensionless numbers and morphological indices representing wing planform and flight state. Specifically, we employed the taper ratio, Reynolds number *Re*, reduced frequency, and lift coefficient *C*_L_. The taper ratio was defined as the ratio of the root chord length *c*_root_ at the 20% of the wing length from wing base to the tip chord length *c*_tip_ at the 80% of the wing length from wing base (Fig. 2):


\begin{eqnarray*}
\textit{Taper}\,\, \textit{ratio} = \frac{{{{C}_{{\mathrm{root}}}}}}{{{{C}_{{\mathrm{tip}}}}}}
\end{eqnarray*}

The Reynolds number (*Re*) was calculated using the representative flight speed *V*, mean chord length $\bar{c}$, fluid density $\rho $, and dynamic viscosity $\mu $ (or kinematic viscosity $\nu $) as


\begin{eqnarray*}
Re = \frac{{{\mathrm{\rho }}Vc}}{\mu } = \frac{{Vc}}{v}
\end{eqnarray*}

The lift coefficient (*C*_L_) was determined from the lift force *L*, flight speed *V*, and wing area *S*


\begin{eqnarray*}
{{C}_L} = \frac{L}{{0.5{\mathrm{\rho }}{{V}^2}S}}.
\end{eqnarray*}

A complete list of species, morphological indices, and original sources for span efficiency is summarized in Table 1.

**Table 1:** Summary of parameters and references.

Species name	Common name	Class	Order	*e*_i_ with tail	*e*_i_ tail 0	*k*	Re	Taper ratio	AR	*V* (m s^−1^)	WBF (Hz)	*m* (g)	*c* (m)	*b* (m)	S (m^2^)	WL (N m^−1^)	Reference (*e*_i_)	Reference (kinematic)
*Apus apus*	Common swift	Aves	Apodiformes	0.618	0.618	0.144	18,848	0.740	10.3	7.90	9.8	42.0	0.037	0.380	1.40 × 10^–2^	29.43	Henningsson et al. (2014)	Henningsson et al. (2008)
*Coloeus monedula*	Jackdaw	Aves	Passeriformes	0.750	0.802	0.083	34,448	0.597	9.8	9.14	4.2	215	0.057	0.564	5.72 × 10^–1^	36.83	KleinHeerenbrink and Hedenström 2016	Spedding (1986)
*Ficedula hypoleuca*	Pied flycatchers	Aves	Passeriformes	0.651	0.902	0.387	12,961	0.579	5.2	4.47	12.2	14.2	0.045	0.235	1.06 × 10^–2^	13.17	Muijres et al. (2012a)	Johansson et al. (2018),
*Sylvia atricapilla*	Blackcap	Aves	Passeriformes	0.546	0.874	0.358	20,108	0.582	5.2	6.78	16.8	16.3	0.046	0.240	1.11 × 10^–2^	14.40	Muijres et al. (2012b)	Wang et al. (2019), Johansson and Hedenström (2009)
*Tyto alba*	Barn owl	Aves	Strigiformes	0.659	0.775	1.667	88,000	0.665	5.1	7.88	24.6	340	0.170	0.870	1.48 × 10^–1^	22.95	Song et al. (2022)	Wolf and Konrath (2015)
*Harmonia axyridis*	Ladybird	Insecta	Coleoptera	0.346	0.820	0.460	141	0.834	11.3	1.00	68.0	0.041	0.002	0.024	5.24 × 10^–5^	0.76	Unpublished data	Le et al. (2013)
*Heliocopris hamadryas*	Dung beetle	Insecta	Coleoptera	0.500	0.620	0.330	6891	0.771	8.4	6.38	40.0	5.888	0.017	0.140	2.14 × 10^–3^	27.59	Johansson et al. (2012)	Johansson et al. (2012)
*Episyrphus balteatus*	Marmalade hoverfly	Insecta	Diptera	0.217	0.719	0.639	265	1.507	4.2	1.80	163.1	0.034	0.002	0.021	2.09 × 10^–5^	1.97	Unpublished data	Walker et al. (2009)
*Eristalis pertinax*	Tapered drone fly	Insecta	Diptera	0.224	0.658	1.112	249	0.966	3.9	1.40	182.9	0.103	0.003	0.025	2.86 × 10^–5^	2.73	Unpublished data	Walker et al. (2009)
*Syrphus ribesii*	Common banded hoverfly	Insecta	Diptera	0.217	0.721	0.592	232	1.150	3.9	1.80	172.3	0.027	0.002	0.018	1.53 × 10^–5^	1.67	Unpublished data	Walker et al. (2009)
*Apis mellifera*	Western honey bee	Insecta	Hymenoptera	0.298	0.856	1.522	219	2.133	3.2	1.20	209.3	0.091	0.003	0.021	2.47 × 10^–5^	3.94	Unpublished data	Altshuler et al. (2005)
*Bombus pascuorum*	Common carder bee	Insecta	Hymenoptera	0.271	0.754	0.797	341	2.404	3.2	1.80	158.2	0.109	0.003	0.022	2.63 × 10^–5^	6.13	Unpublished data	Dudley and Ellington (1990)
*Bombus terrestris*	Buff-tailed bumblebee	Insecta	Hymenoptera	0.293	0.872	0.937	500	2.012	3.1	1.80	126.7	0.243	0.004	0.032	5.49 × 10^–5^	7.02	Unpublished data	Dudley and Ellington (1990)
*Vespula vulgaris*	Common wasp	Insecta	Hymenoptera	0.339	0.884	0.533	261	2.503	4.4	1.80	138.2	0.088	0.002	0.022	2.14 × 10^–5^	2.38	Unpublished data	Dudley and Ellington (1990)
*Deilephila elpenor*	Elephant hawkmoth	Insecta	Lepidoptera	0.602	0.932	1.106	1199	0.588	5.5	1.45	41.0	0.850	0.012	0.068	8.51 × 10^–4^	9.76	Henningsson and Bomphrey (2013)	In-house data
*Hemaris fuciformis*	Broad-bordered bee hawkmoth	Insecta	Lepidoptera	0.315	0.488	0.887	548	0.658	6.7	1.38	65.0	0.200	0.006	0.040	2.40 × 10^–4^	8.19	Henningsson and Bomphrey (2013)	In-house data
*Hyles euphorbiae*	Spurge hawkmoth	Insecta	Lepidoptera	0.506	0.785	0.783	1778	0.581	5.0	2.13	42.0	0.887	0.013	0.064	7.98 × 10^–4^	10.95	Henningsson and Bomphrey (2013)	In-house data
*Macroglossum stellatarum*	Hummingbird hawkmoth	Insecta	Lepidoptera	0.457	0.709	0.958	834	0.572	5.1	1.50	55.0	0.320	0.008	0.043	3.72 × 10^–4^	8.88	Henningsson and Bomphrey (2013)	In-house data
*Manduca sexta*	Tobacco hawkmoth	Insecta	Lepidoptera	0.462	0.715	0.386	3689	0.623	6.0	3.17	22.0	1.500	0.018	0.106	1.70 × 10^c^	7.82	Henningsson and Bomphrey (2013)	In-house data
*Sphinx ligustri*	Privet hawkmoth	Insecta	Lepidoptera	0.409	0.634	0.689	1894	0.567	5.6	1.80	25.0	1.480	0.016	0.089	1.40 × 10^–3^	10.20	Henningsson and Bomphrey (2013)	In-house data
*Aeshna grandis*	Brown hawker	Insecta	Odonata	0.740	0.865	0.908	1823	0.761	4.5	1.37	19.5	0.720	0.020	0.092	5.90 × 10^–3^	1.20	Bomphrey et al. (2016)	In-house data
*Aeshna mixta*	Migrant hawker	Insecta	Odonata	0.444	0.610	0.741	1541	0.728	4.6	1.35	18.4	0.550	0.017	0.081	4.61 × 10^–3^	1.17	Bomphrey et al. (2016)	In-house data
*Anax imperator*	Emperor dragonfly	Insecta	Odonata	0.443	0.503	1.064	1603	0.715	5.2	1.30	23.4	1.150	0.019	0.098	6.54 × 10^–3^	1.73	Bomphrey et al. (2016)	In-house data
*Calopteryx splendens*	Banded demoiselle	Insecta	Odonata	0.346	0.442	0.713	981	1.585	4.2	1.10	18.2	0.119	0.014	0.057	2.16 × 10^–3^	0.57	Bomphrey et al. (2016)	In-house data
*Enallagma cyathigerum*	Common blue damselfly	Insecta	Odonata	0.250	0.339	0.296	351	2.321	6.7	1.00	17.7	0.030	0.005	0.036	5.33 × 10^–4^	0.56	Bomphrey et al. (2016)	In-house data
*Sympetrum striolatum*	Common darter	Insecta	Odonata	0.587	0.643	0.724	788	0.766	4.9	1.00	19.1	0.190	0.012	0.058	2.52 × 10^–3^	0.75	Bomphrey et al. (2016)	In-house data
*Schistocerca gregaria*	Desert locust	Insecta	Orthoptera	0.530	0.795	0.200	4400	0.538	6.6	3.30	20.0	1.957	0.011	0.096	2.01 × 10^–3^	21.13	Henningsson and Bomphrey (2012)	Henningsson and Bomphrey (2012)
*Glossophaga soricina*	Pallas’s long-tongued bat	Mammalia	Phyllostomidae	0.347	0.816	0.408	10,405	0.613	6.2	4.30	14.9	9.800	0.038	0.232	8.70 × 10^–3^	11.05	Muijres et al. (2011a, 2011b); Håkansson et al. (2017)	Wolf et al. (2010)
*Leptonycteris yerbabuenae*	Southern long-nosed bat	Mammalia	Phyllostomidae	0.469	0.790	0.498	10,912	0.508	7.0	3.27	11.0	22.6	0.047	0.329	1.50 × 10^–2^	14.25	Muijres et al. (2011a, 2011b), Håkansson et al. (2017)	Von Busse et al. (2012)
*Plecotus auritus*	Brown long-eared bat	Mammalia	Vespertilionidae	0.322	0.400	0.508	9650	0.694	5.9	3.00	10.0	9.10	0.049	0.287	1.38 × 10^–2^	6.50	Johansson et al. (2016), Håkansson et al. (2017)	Norberg (1970, 1976)

*Note:* The following abbreviations and symbols are used: ei (with tail), span efficiency including integration tail length; ei (tail 0), span efficiency without integration tail length; k, reduced frequency; Re, Reynolds number; AR, aspect ratio; V, flight speed (m s^−1^); WBF, wing beat frequency (Hz); m, body mass (kg); c, wing mean chord length (m); b, wing span from one tip to another (m); S, wing area (m^2^); WL, wing loading (N m^−2^).

### Span efficiency

Span efficiency (*e*_i_) is an aerodynamic index quantifying how closely the actual lift distribution approximates the ideal elliptical distribution from the perspective of induced power. Generally, it is defined as the ratio of the induced power corresponding to the ideal elliptical lift distribution (*P*_i, ideal_) to the actual induced power estimated from wake measurements (*P*_i, real_). Using the spanwise downwash velocity distribution *U*(*z*) obtained from the wake, *e*_i_ is expressed as


\begin{eqnarray*}
{{e}_{\mathrm{i}}} = \frac{{{{P}_{{\mathrm{i,{\rm ideal}}}}}}}{{{{P}_{{\mathrm{i,{\rm real}}}}}}} = \frac{4}{{\pi {{b}^2}}}\frac{{{{{\left( {\smallint _{ - \frac{b}{2}}^{\frac{b}{2}}U\left( z \right)\sqrt {{{b}^2} - 4{{z}^2}{\rm d}z} } \right)}}^2}}}{{\smallint _{ - \frac{b}{2}}^{\frac{b}{2}}{{U}^2}\left( z \right)\sqrt {{{b}^2} - 4{{z}^2}{\rm d}z} }},
\end{eqnarray*}

where *b* denotes the wingspan and *z* is the spanwise coordinate. A value of *e*_i_ closer to unity indicates a higher efficiency, approaching the theoretical ideal. In experimental wake measurements, the downwash velocity distribution *U*(*z*) is extracted from the measured flow field. Owing to the square term, any non-uniformity in this distribution manifests as a relative increase in the denominator (actual induced power), thereby reducing the efficiency. While the integration limits in the equation typically correspond to the spanwise distance between the vortex centres or the outer edges of the wingtip vortices, the specific selection of this integration domain (tail) varies across existing literature. To evaluate fairly how these methodological discrepancies affect the estimation of *e*_i_ we utilized CFD simulations to assess sensitivity of the results to variation in the integration domain, establishing the foundation for the correction framework described in the following section. We also assessed sensitivity to measurement noise and downstream distance of the measurement plane.

### Computational fluid dynamics

The primary objective of the CFD analysis in this study was not to use models that replicate the exact morphology of individual species, but rather to establish a universal correction framework based on a ground truth. This framework aims to standardize existing experimental data by quantifying the systematic biases introduced by variations in measurement distance, experimental noise, and analysis protocols. We used a standard elliptical wing model (NACA 0012 aerofoil) with a span of 0.1 m and an aspect ratio of 6.5. The angle of attack was set to 5° to avoid stall. Free-stream velocities were set between 3.3 and 10 m/s, corresponding to Reynolds numbers (*Re*) based on mean chord length of approximately 3300 (cf. desert locust) to 10,000 (cf. birds). The computational domain was defined as a cubic volume of 0.35 × 0.35 × 0.35 m. Boundary conditions were set as uniform inflow at the inlet, no-slip condition on the wing surface, and zero-gradient (Neumann) conditions at the remaining boundaries.

Numerical simulations were performed using OpenFOAM (v2212, OpenCFD Ltd.). We used simpleFoam, a steady-state solver for incompressible Navier–Stokes equations, a framework whose reliability for bio-inspired aerodynamic evaluations has been established in our previous work (Kagawa et al. 2025). Given the relatively low Reynolds numbers, no turbulence model was implemented. In this regime, omitting turbulence models avoids the artificial diffusion often introduced by standard RANS models, preserving the natural spatial attenuation of the wake (Shyy et al. 2009). While this steady laminar approach may not capture fine, unsteady vortex structures arising from shear layer interactions, it effectively resolves the macroscopic tip vortex roll-up and downwash distribution. This provides an idealized, controlled baseline essential for systematically quantifying spatial integration biases in PIV analyses. The computational mesh was generated using ANSYS ICEM CFD. We constructed a hybrid mesh consisting of prism layers near the wing surface to resolve the boundary layer, and unstructured tetrahedral cells in the rest of the domain. The near-wall mesh resolution was determined based on the empirical boundary layer thickness (0.1*c*/$\surd $*Re*), ensuring sufficient resolution even at the lower Re. Crucially, given our focus on wake topology and *e*_i_ estimation, we locally refined the mesh in the wake region extending beyond 20 chord lengths (20*c*) downstream (typical cell size of 0.0066 $\bar{c}$) to minimise numerical diffusion. The steady-state simulations converged uniformly, with residuals for all equations dropping below 2 × 10^–5^ and aerodynamic forces reaching stable values. Grid independence studies (verification) are detailed in Table 2. At both Reynolds numbers tested, the computed lift and drag coefficients were higher with the coarsest mesh (Coarse3) but demonstrated clear convergence as the grid resolution increased toward the finest mesh (designated as the “Normal” mesh). Therefore, we concluded that the “Normal” mesh provided sufficient spatial resolution for our simulations.

**Table 2:** Grid dependence of drag and lift coefficients at Reynolds numbers of 3300 and 10,000.

Model	Total cells	C_D_*Re* = 3300	C_L_*Re* = 3300	C_D_*Re* = 10,000	C_L_*Re* = 10,000
Normal	45,353,235	0.0748	0.231	0.0515	0.287
Coarse1	27,001,570	0.0758	0.245	0.0520	0.284
Coarse2	17,657,947	0.0770	0.259	0.0510	0.281
Coarse3	12,990,484	0.0783	0.273	0.0508	0.290

Span efficiency correction factors were defined as the ratio of the efficiency calculated under ideal standardized conditions to that obtained under particular experimental scenarios. Specifically, we identified and quantified three potential sources of bias.

1. Streamwise distance: We systematically varied the location of the measurement plane from the near-wake to 20 chord lengths downstream (20*c*) to quantify the decay in *e*_i_ caused by wake roll-up and diffusion.
2. Experimental noise: To simulate PIV measurement errors, synthetic noise was superimposed on the computed velocity field data. Because downwash velocities in biological flight are typically smaller than the freestream velocity, standard uncertainties (e.g., ∼2% of the freestream; Raffel et al. 2018) yield a low signal-to-noise ratio for the induced velocity components. Therefore, we generated random Gaussian noise, scaled the maximum absolute amplitude to 35% of the mean downwash velocity, and added it to each local velocity vector along the transect.
3. Integration domain: We evaluated the sensitivity of *e*_i_ to the spanwise extent of the integration domain (the tail)—specifically how far beyond the wingtip vortex axis the analysis extends—by applying different tail lengths used by various research groups to the CFD-generated velocity fields.

Note that while parameters such as streamwise distance (1) and the integration domain (3) are fundamentally user-defined in individual experiments, practical constraints in biological PIV, such as wind tunnel dimensions, animal safety, and limitations in camera field-of-view, often prevent their standardization across different studies. Consequently, when compiling literature data for a meta-analysis, variations in these parameters act as sources of systematic cross-study bias that require correction.

### Theoretical estimate of the span efficiency

Classical LLT (Anderson 2011) estimates the induced drag based on the spanwise circulation distribution of a finite wing, quantifying the deviation from the ideal elliptical circulation as a loss in efficiency. As a fundamental classical framework, LLT is widely utilized for evaluating the aerodynamic performance of wings. In this study, we employed LLT to calculate the morphology-based span efficiency (*e*_i, gliding_) derived solely from the wing and body planform, assuming a steady gliding condition. By comparing this morphological baseline with the experimentally determined flapping efficiency (*e_i,real_*) obtained from wake measurements, we evaluated the divergence between the baseline performance dictated by wing planform alone and the actual effective efficiency realized during flapping flight.

**Fig. 3:**
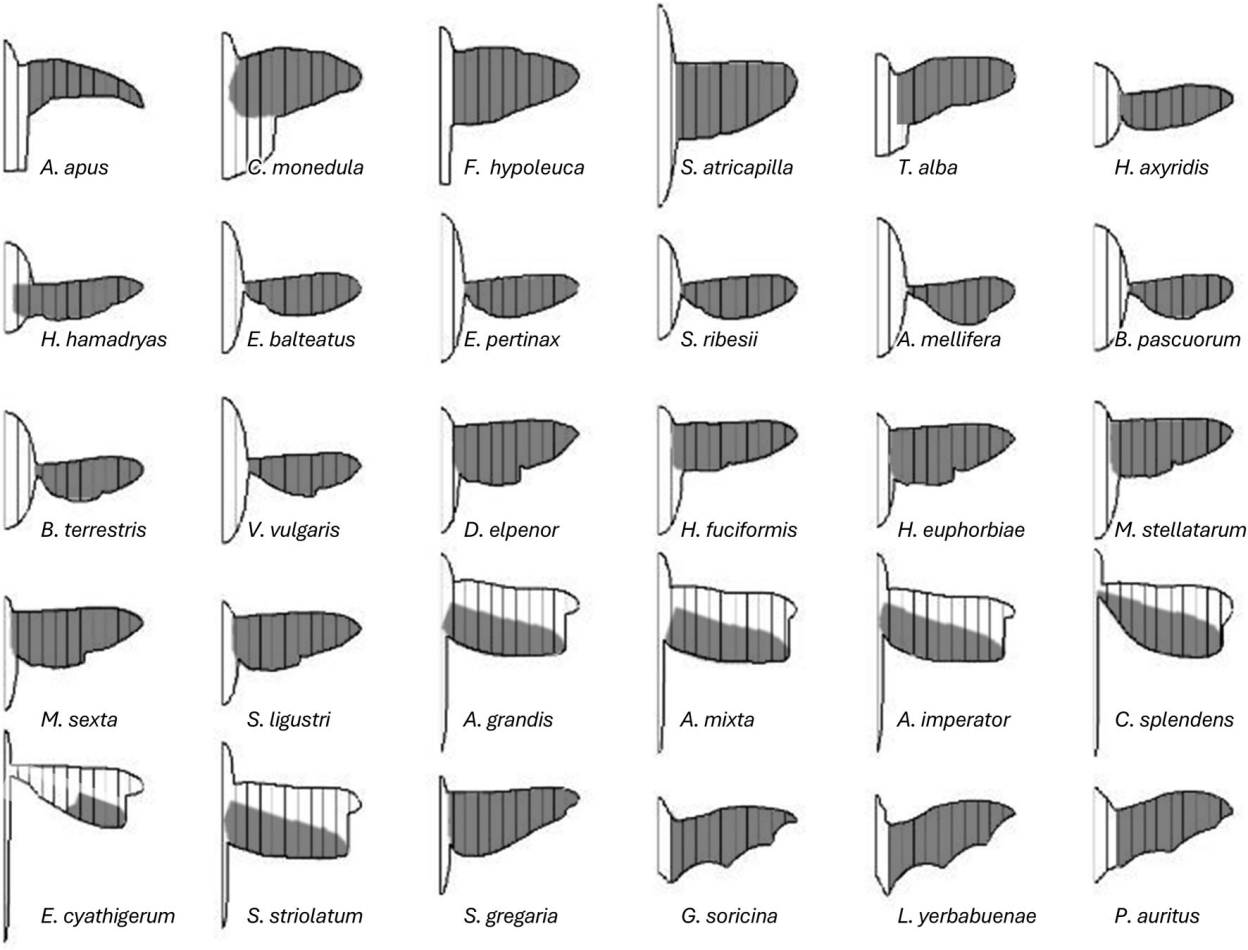
Wing planforms and blade elements used for theoretical span efficiency estimation. The wing and body outlines of the target insects, birds, and bats are shown, with only the right half depicted for simplicity. The full planforms were utilized for the gliding LLT analysis. The grey-shaded subregions represent the effective wing areas used for the BEMT analysis, which assumes rotation around the wing root/hinge. For Odonata, only the hindwings were considered for this rotational evaluation.

Prior to the LLT analysis, we performed geometric extraction and alignment of the wing planforms from the collected images (Fig. 3). Specifically, the wing and body planform outlines were segmented. The body was approximated as an ellipse to standardize the fuselage influence, while the wings were digitally rotated to maximize the horizontal span. This process generated a consistent wing-body configuration input for the subsequent numerical analysis and comparison.

We used Blade Element Momentum Theory (BEMT; Stepniewski and Keys 1984) to estimate the span efficiency when revolving. This classical framework combines the evaluation of local aerodynamic forces on spanwise blade elements with the momentum balance of the induced flow field to estimate lift generation and induced losses. It is a widely accepted classical method for estimating baseline aerodynamic performance under idealized steady rotary kinematics. The primary objective of introducing BEMT was to isolate the morphological component of span efficiency. Experimental efficiency values derived from wake measurements inevitably entangle the effects of static wing planform with complex kinematic factors (e.g., stroke amplitude, flexibility/deformation) and unsteady aerodynamic mechanisms. BEMT allows us to strip away these kinematic complexities and evaluate the baseline performance dictated by shape alone. Specifically, we utilized the chord distribution *c*(*r*) extracted from the wing planforms as the primary input. Assuming a simplified quasi-steady rotary motion to represent the flapping stroke, we iteratively solved for the local lift and induced inflow velocity at each blade element.

From the resulting spanwise distribution of induced velocity (downwash), we calculated a morphology-based span efficiency (*e_i,rotary_*) that reflects solely the influence of static wing planform. By comparing this morphology-based estimate against the experimentally determined values (*e_i,real_*), we quantified the correlation and divergence between the two. This approach allowed us to assess the extent to which wing shape contributes to the efficiency differences observed in wind tunnel tests and to verify the feasibility of predicting aerodynamic performance based on planform information alone.

### Phylogenetic analyses

The primary objective of this study is to evaluate the covariance between span efficiency (*e*_i_) and morphological or kinematic indices while explicitly accounting for evolutionary non-independence—the tendency of closely related species to share similar traits. Since species data cannot be treated as independent data points, applying standard regression or correlation analyses risks inflating type I errors (overestimation of significance). To address this, we employed PCMs to model the covariance structure based on evolutionary history. Statistically, we applied: (1) phylogenetic generalized least square (PGLS) for univariate association assessments (Symonds and Blomberg 2014); and (2) Phylogenetic Partial Least Squares (Phylo-PLS) for evaluating multivariate covariance structures (Adams and Collyer 2016). To implement these analyses, we constructed a comprehensive time-calibrated phylogenetic tree by integrating and pruning published chronograms. For birds and bats, we utilized recent comprehensive phylogenies that covered the target species tips (Birds: Stiller et al. 2024; Bats: Hao et al. 2024). For insects, we constructed a composite tree using the class-level phylogeny of (Misof et al. 2014) as the backbone. Onto this backbone, we grafted higher-resolution sub-trees for specific orders: Odonata (Carvalho et al. 2022), Hymenoptera (Peters et al. 2017; Santos Júnior et al. 2022), Lepidoptera (Kawahara et al. 2009; Huang et al. 2022), Diptera (Wu et al. 2024), and Coleoptera (Cai et al. 2022). From these source trees, we extracted the relevant clades, pruned non-target species, and integrated them hierarchically using a cut-and-paste approach to generate the final consensus tree used for the analyses.

## Results

### Standardization of span efficiency

**Fig. 4:**
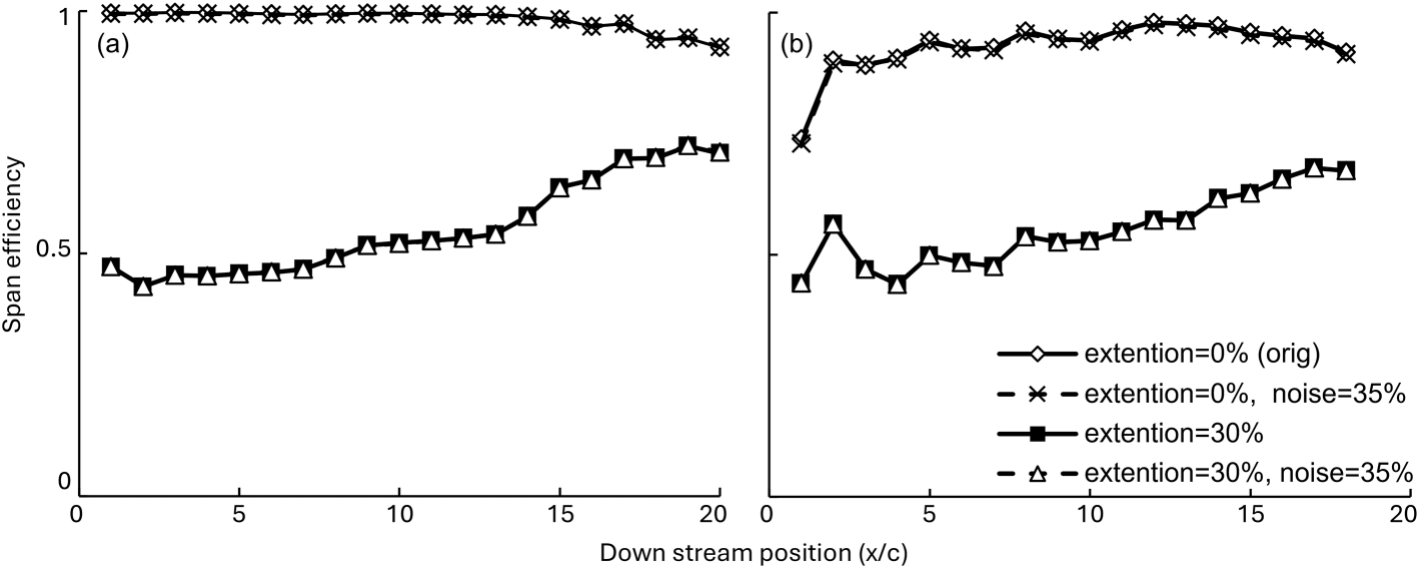
Effects of downstream measurement distance, experimental noise, and integration tail length on span efficiency obtained via CFD. Results are shown for two Reynolds numbers: (A) *Re* = 3300 and (B) *Re* = 10,000. The legend denotes the extension of the integration range (tail length) beyond the wingtips (0% or 30%) and the level of superimposed noise (0% or 35%).

Numerical simulations revealed that among the factors examined—measurement position, measurement noise, and the tail length added to the transect—tail length had the most significant impact on span efficiency (Fig. 4). At Reynolds numbers (*Re*) of 3300 and 10,000, the influence of measurement noise (35%) was found to be negligible even though a high level of Gaussian noise was added to the spatial downwash distribution. This robustness likely stems from the fact that stochastic fluctuations effectively cancel out during the spanwise integration process. In contrast, increasing the tail length from 0% to 30% resulted in a marked decrease in span efficiency. Specifically, at a standard measurement position of approximately 8 chord lengths downstream, span efficiency decreased by 51% at *Re* = 3300 and by 36% at *Re* = 10,000 when the tail was included. In the absence of a tail (0% tail length; transects end at the wing tip vortex core axis), the effect of the distance between the wing and the measurement plane was relatively minor and remained essentially constant. The slight decrease in efficiency observed when the measurement plane was too close at higher Reynolds numbers is likely attributable to variation in downwash distribution due to unsteady vortex shedding caused by flow separation from the wing surface. Conversely, with a tail length of 30%, span efficiency increased monotonically as the measurement position moved further downstream. This trend suggests that complex flow structures captured by the inclusion of the tail, such as wingtip vortices, are gradually smoothed by viscous dissipation as they propagate downstream. Since the measurement positions employed in previous studies generally fall within the range of 2–11 chord lengths, the influence of measurement position can be considered minimal within this range.

Based on these findings, we standardized the dataset by neglecting the effects of noise and measurement position. For measurements where no tail was added to the integration domain, we applied a correction factor: span efficiency was reduced by 51% for insects (based on the *Re* = 3300 case) and by 36% for birds and bats (based on the Re = 10,000 case).

### Phylogenetic analyses

**Fig. 5:**
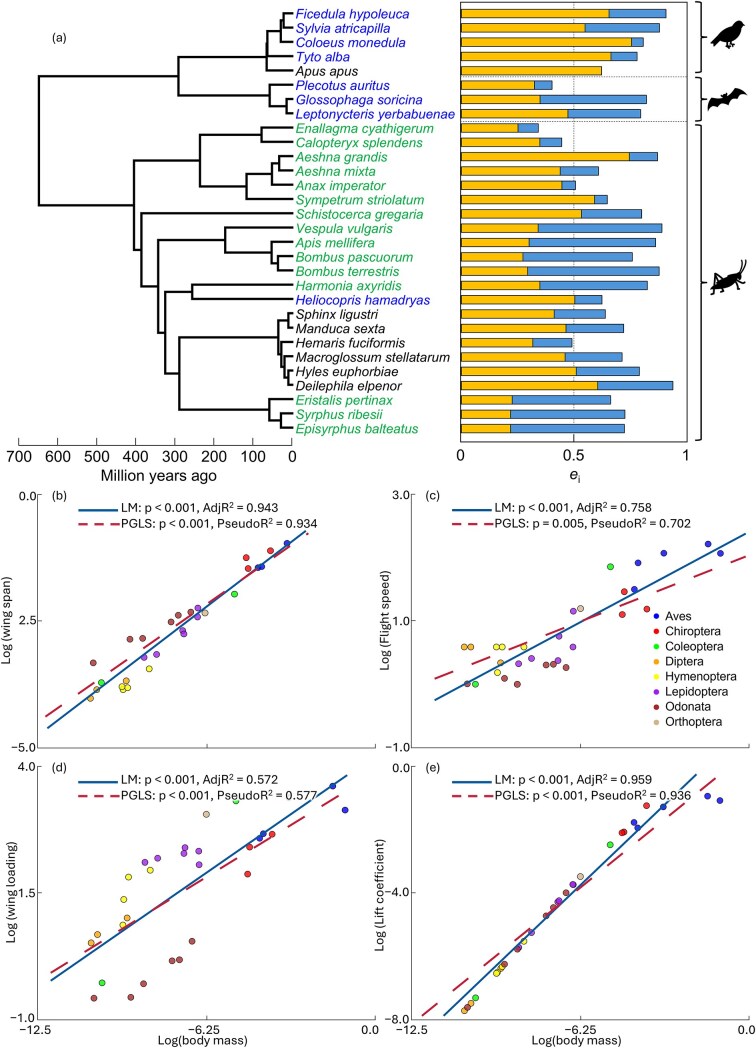
Phylogenetic relationships, span efficiency, and scaling of flight parameters. (A) The constructed time-calibrated phylogenetic tree and the corresponding span efficiency (*e*_i_) for each species. The blue and yellow bars represent span efficiency calculated without integration tails (downwash integrated strictly between the wingtip vortex cores) and with the tail (including velocity vectors outboard of the vortex cores), respectively. (B–E) Scaling relationships showing log-transformed body mass against (B) wingspan, (C) flight speed, (D) wing loading, and (E) lift coefficient. Solid blue and dashed red lines represent standard LM and PGLS regressions, respectively.

Based on previous studies, we constructed a phylogenetic tree comprising 30 species (Fig. 5A). The dataset covered a broad range of body sizes: body mass ranged from a hoverfly (*Syrphus ribesii*) weighing 0.027 g to a barn owl (*Tyto alba*) weighting 0.34 kg; wing lengths ranged from 17.8 mm (*S. ribesii*) to 0.87 m (*T. alba*). In this dataset, as expected, body mass and wingspan exhibited a strong positive correlation (Fig. 5B; linear model [LM]: *P* < 0.001, Adj. *R*^2^ = 0.943; PGLS: *P* < 0.001, Adj. *R*^2^ = 0.934). Flight speed also increased with body size within this dataset (Fig. 5C). Similarly, both wing loading and lift coefficient (*C*_L_) scaled significantly with body mass (Fig. 5D and E); the increase in wing loading is expected from dimensional scaling analysis, as it is defined as weight divided by wing area. Correcting for the integration domain tail generally resulted in a reduction of span efficiency, although the magnitude of this reduction varied by species (Fig. 5A, right). The mean corrected span efficiency (*e*_i_) was 0.645 ± 0.074 for birds, 0.379 ± 0.079 for bats, and 0.400 ± 0.141 for insects. Standard statistical analysis (ANOVA followed by Tukey HSD post hoc test) shows that birds demonstrate significantly higher span efficiency compared with insects (*P* = 0.0019) and bats (*P* = 0.0237), while no significant difference was found between insects and bats (*P* = 0.964). In contrast, phylogenetic ANOVA (Garland et al. 1993; 10,000 simulations) revealed no statistically significant differences among the groups (*P* = 0.485).

**Fig. 6:**
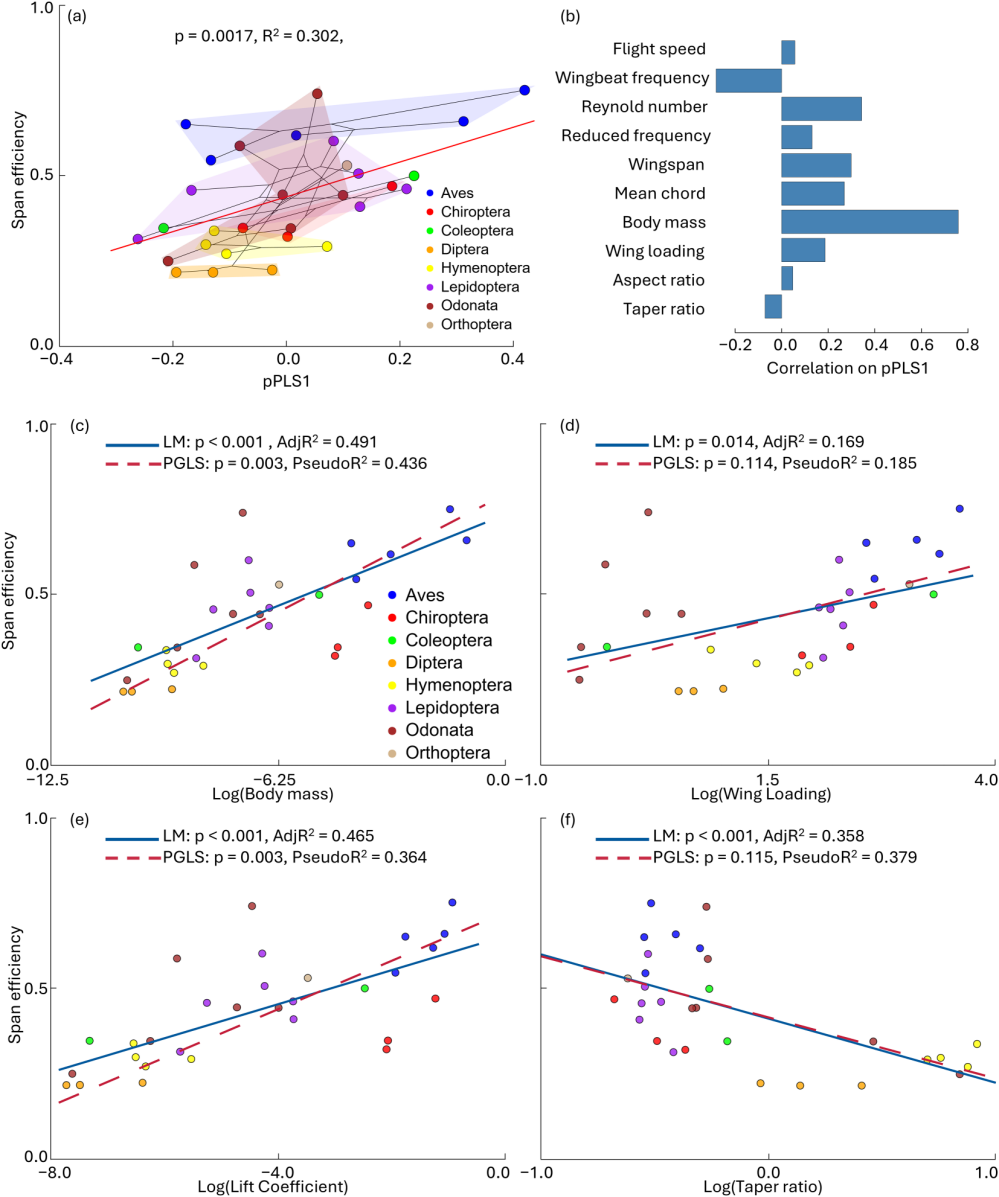
Correlations between span efficiency and morphological/kinematic parameters. (A) Relationship between the first component of the phylogenetic partial least squares analysis (pPLS1) and measured span efficiency. (B) Contribution of each parameter to the pPLS1 axis. (C–F) Relationships between span efficiency and log-transformed (C) body mass, (D) wing loading, (E) lift coefficient, and (F) taper ratio. Solid blue and dashed red lines in panels (C–F) represent standard LMs and phylogenetic generalized least squares (PGLS) regressions, respectively.

Our standardized analysis reveals that span efficiency is strongly influenced by body size. To disentangle the covariates best explaining the variation in span efficiency, we performed a phylogenetic Partial Least Squares (phylo-PLS) analysis. The first component of this analysis (pPLS1) showed a significant correlation with span efficiency (*P* < 0.005, *R*^2^ = 0.284; Fig. 6A). Decomposing the structure of pPLS1 reveals that body mass has the largest loading weight, indicating it is the dominant factor (Fig. 6B). Indeed, a direct regression of span efficiency against body mass (Fig. 6C) yielded significant correlations in both LM and PGLS analyses. Consequently, variables that scale with mass—specifically wingspan and wing loading (see Fig. 5) and Reynolds number (*Re*)—also exhibited high contributions to pPLS1. Kinematic parameters also played a significant role. Flapping frequency showed a strong correlation with body mass (LM: *P* < 0.001, Adj. *R*^2^ = 0.508; PGLS: *P* < 0.001, Adj. *R*^2^ = 0.481) and was a major contributor to the pPLS1 vector. Similarly, reduced frequency, which is derived from frequency and wing size, was correlated with body mass in the raw data (LM: *P* = 0.003, Adj. *R*^2^ = 0.241), although this correlation was not significant after phylogenetic correction (PGLS: *P* = 0.756, Adj. *R^2^* = −0.103). A similar pattern was observed for wing loading; while it showed a significant correlation in the standard analysis (LM: *P* = 0.014), this relationship was not significant after phylogenetic correction (PGLS: *P* = 0.114; Fig. 6D). Conversely, the lift coefficient (*C*_L_) was significantly correlated with span efficiency in both the standard (LM: *P* < 0.001) and phylogenetic (PGLS: *P* = 0.003) analyses (Fig. 6E).

Among the dimensionless morphological parameters independent of body mass, taper ratio showed the most significant contribution to predicting span efficiency (Fig. 6F). Specifically, wings with a smaller taper ratio—reflecting a shorter chord length at the wingtip relative to the root—exhibited higher span efficiency. As noted in the Introduction, an elliptical planform is theoretically ideal for maximizing span efficiency, as it generates a uniform downwash distribution and suppresses wingtip vortices. Conversely, a wing with a large chord length near the tip (i.e., a high taper ratio) generates excessive lift in the tip region. This effect is exacerbated in the case of flapping, because the tip region is also moving at a higher velocity than the root. Similarly, unsteady mechanisms such as LEV may enhance lift during flapping, but can introduce nonuniformity into the downwash, reducing efficiency. This causes the lift distribution to deviate from the ideal ellipse, resulting in a non-uniform downwash distribution. Therefore, the observed negative correlation between taper ratio and span efficiency is physically consistent with aerodynamic theory. The influence of aspect ratio was found to be relatively minor.

### Lifting-line theory and blade element and momentum theory analysis

**Fig. 7:**
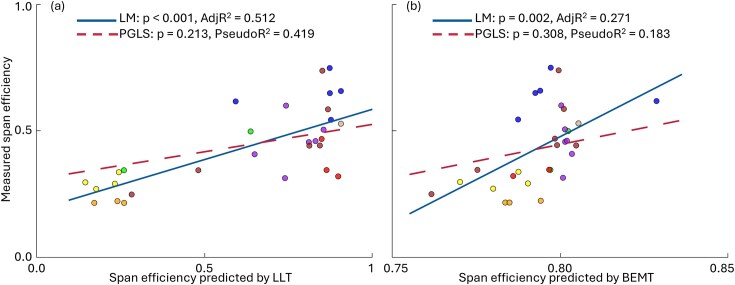
Correlations between theoretical and experimentally measured span efficiency. (A) Relationship between the measured span efficiency and the theoretical span efficiency for gliding flight, predicted by LLT. (B) Relationship between the measured span efficiency and the theoretical span efficiency for rotational motion, predicted by BEMT. Solid blue and dashed red lines represent standard LM and PGLS regressions, respectively.

Theoretical span efficiency derived from wing planform—calculated for both gliding and rotary wing scenarios—exhibited a significant positive correlation with measured span efficiency in the LM analysis (*R*^2^ = 0.419, *P* < 0.001 for LLT; *R*^2^ = 0.229, *P* = 0.005 for BEMT; Fig. 7). However, when accounting for phylogenetic nonindependence, this significance disappeared in the PGLS analysis (*P* = 0.339 for LLT; *P* = 0.372 for BEMT). This statistical discrepancy indicates that the correlation observed in the LM is primarily driven by phylogenetic structure among the major taxonomic groups rather than independent evolutionary events.

## Discussion

This study presents the first meta-phylogenetic analysis of span efficiency—a key determinant of flight performance—across insects, birds, and bats, three groups that independently evolved powered flight. While span efficiency has been individually measured for most of these species in previous studies, methodological inconsistencies in measurement and evaluation across research groups have hindered a unified, comprehensive analysis. In this study, we established a framework for standardized comparison by ensuring the consistency of existing datasets through corrections based on numerical simulations. Our results revealed a robust positive correlation between span efficiency and body size (mass) that is independent of phylogeny (significant in both LM and PGLS). In contrast, correlations with wing morphology (taper ratio) and theoretical predictions based on wing contours disappeared when phylogenetic relationships were accounted for (significant only in LM). These findings suggest that biological flight efficiency is governed by the interplay of two distinct factors: universal physical constraints associated with size scaling, and lineage-specific adaptive strategies—such as kinematics and wing structure—uniquely acquired by each clade.

The robust positive correlation between size and span efficiency observed in this study (significant in PGLS) suggests that this trend is a result of strong evolutionary selection pressure to mitigate the increase in induced drag associated with larger body size. Generally, wing loading (*Q* = *W*/*S*) scales positively with body size (Fig. 5D). Assuming that lift balances weight in steady flight, the required lift coefficient (*C*_L_) is proportional to wing loading (*C*_L_  $\propto$  *Q*); therefore, larger organisms with higher wing loading are compelled to fly at higher *C*_L_ to support their weight. Indeed, a lift coefficient scales positively with body mass (Fig. 5E). It is important to note here that the induced drag coefficient—the drag penalty associated with lift generation—is proportional to the square of *C_L_* and inversely proportional to span efficiency (*e*_i_) (*C*_Di_  $\propto$*C*_L_*^2^*)/e (Spedding and McArthur 2010). Consequently, for larger organisms obligated to fly at high *C*_L_, any decrease in span efficiency would lead to a drastic increase in induced drag, resulting in prohibitive flight costs. In contrast, smaller organisms possess relatively lower wing loading and operate at lower Reynolds numbers (Tennekes 2009), where the contribution of induced drag to total drag is smaller relative to viscous (profile) drag. Indeed, it has been reported that even in birds, tail posture is adjusted to minimise viscous drag rather than induced drag (Usherwood et al. 2020). Thus, the selection pressure to optimize span efficiency is likely less intense compared to larger species. We infer that this size-dependent sensitivity to induced drag costs acts as a physical driver, forcing larger species to converge toward higher span efficiency.

The discrepancy between the significant correlation of wing morphology with span efficiency in the LM analysis and its disappearance in the PGLS analysis implies that each lineage has evolved unique strategies to maximise efficiency by optimizing non-morphological factors—such as flapping kinematics and wing flexibility—within their specific morphological constraints. Indeed, insects, birds, and bats employ fundamentally different aerodynamic mechanisms. Insects actively exploit LEV via large flapping amplitudes, relying primarily on kinematic control and passive twisting to adjust lift distribution (Sane 2003). In contrast, vertebrate fliers (birds and bats) share the capability for dramatic active wing morphing (Lentink et al. 2007; Yu and Guan 2015); however, distinct differences exist in their aerodynamic characteristics. As demonstrated by Hubel et al. (2016), bats achieve complex control over camber and twist dynamically within a wingbeat cycle using their articulated, elongated digits. However, Muijres et al. (2012) noted that bats generally exhibit lower span efficiency compared with birds, attributing this discrepancy to structural differences in the wing. Whereas avian feathered wings maintain a relatively continuous surface during flapping—allowing for a near-ideal lift distribution—the bat wing membrane is segmented by the digital skeleton. This structural segmentation promotes the shedding of multiple small vortex rings into the wake, thereby increasing induced drag. Consequently, even when the static wing morphologies (and hence the theoretical predictions) appear similar, the underlying kinematic controls and resultant wake structures can differ fundamentally between lineages. This likely explains why the explanatory power of morphology alone diminishes when phylogenetic relationships are accounted for in the PGLS analysis.

It is important to acknowledge certain limitations regarding sampling distribution and the scope of analysis in this study. First, the dataset is not well distributed throughout species capable of powered flight and is subject to taxonomic bias. Although insects represent the most speciose group in the animal kingdom, this analysis does not include very small insects such as fruit flies and mosquitoes (Diptera). Furthermore, the number of vertebrate data points is limited relative to their actual diversity. While avian wing morphology is varied (Rader and Hedrick 2023) our dataset does not envelop this diversity. For instance, there are no pelagic seabirds with particularly high aspect ratios, such as albatrosses. Similarly, the dataset does not fully encompass the diversity of bat sizes and wing morphologies (Norberg and Rayner 1987). These gaps are due to technical and cost constraints of experimental setups, the time it takes for capture and processing, and the limited number of labs equipped to make measurements. Wake measurements for minute insects present significant challenges regarding tethering and high-resolution imaging of fine structures. Conversely, for large birds, physical barriers arise from the size limitations of wind tunnel test sections, such as blockage effects and laser power. Consequently, the disappearance of the correlation between taper ratio (or theoretical span efficiency) and measured efficiency in the PGLS analysis may partly stem from the limited number of species within each clade, which could have obscured the phylogenetic signal. Second, there is a lack of parameters representing the temporal evolution of wing morphology and motion. As discussed, differences in wing deformation and flapping kinematics are suggested to be primary drivers of efficiency differences among lineages. However, due to heterogeneity of the dataset, we were unable to explicitly incorporate kinematic variables, such as flapping amplitude, as potential explanatory variables in this meta-analysis. Therefore, future research should aim to accumulate new experimental data filling these taxonomic gaps at the extremes of size, shape and movement, supported by innovations in experimental technology. Furthermore, constructing comprehensive models that integrate both morphology and kinematics will be essential for a deeper understanding of efficiency and inefficiency, in biological flight.

**Fig. 8:**
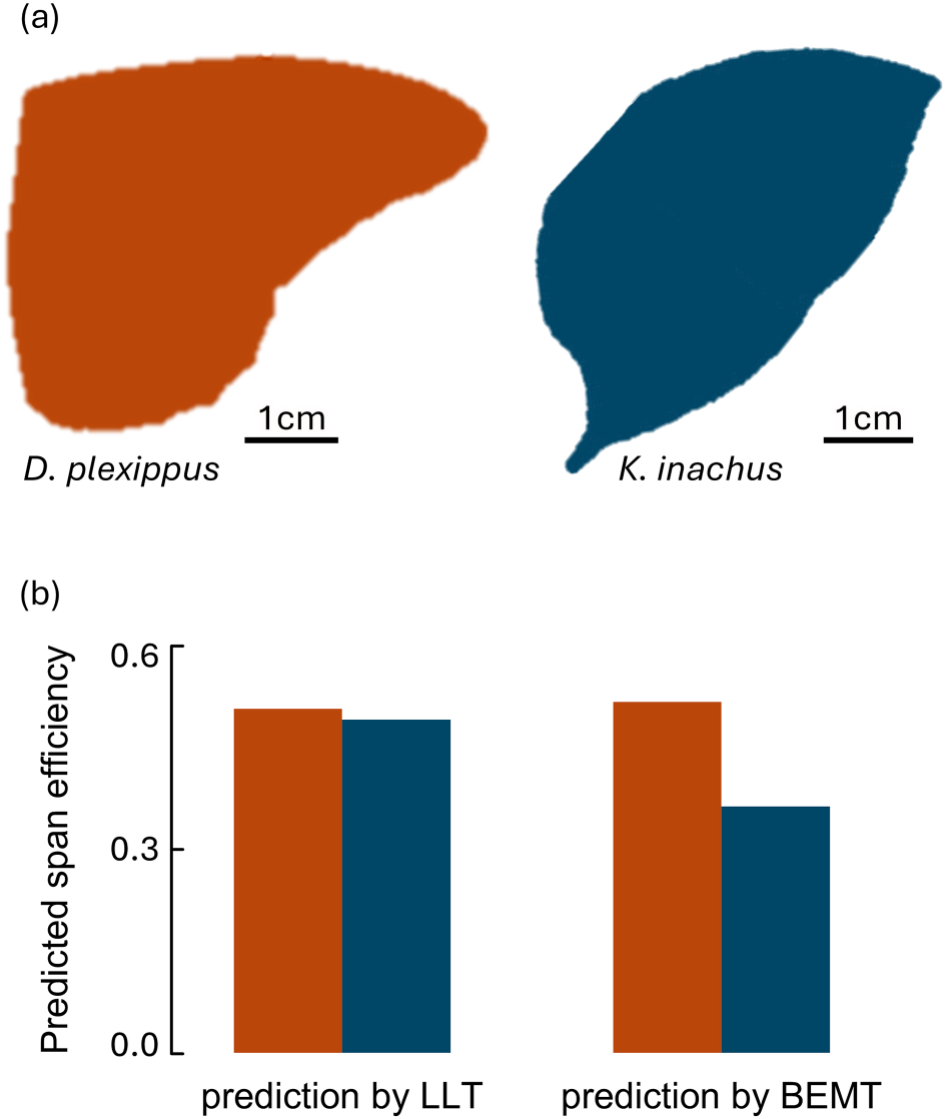
Aerodynamic cost of butterfly mimicry evaluated by LLT and BEMT. (A) Wing outlines of the monarch butterfly (*D. plexippus*; Ortega Ancel et al. 2017) and the orange oakleaf butterfly (*K. inachus*; Wang et al. 2022). (B) Predicted experimental span efficiencies for the monarch butterfly (orange) and the oakleaf butterfly (blue). The predictions are based on lifting-line theory (LLT, left) and BEMT, right calculations, converted using the standard LM derived in this study.

The significant correlation observed in the LM analysis between wing morphology (taper ratio and theoretical estimates) and measured span efficiency corroborates the view that biological wings have generally evolved in accordance with aerodynamic principles. As dictated by classical lifting-line theory, an elliptical lift distribution is ideal for minimising induced drag. Regardless of whether the flight mode is gliding or flapping, a tapered wing planform is geometrically advantageous for approximating this distribution. The prevalence of tapered wings across diverse taxa is likely a direct reflection of this physical imperative. However, substantial divergence in taper ratios exists even among closely related species, implying trade-offs between aerodynamic efficiency and other ecological factors. For instance, Bomphrey et al. (2016) noted that, since within the Odonata, damselflies possess broader wingtips compared with dragonflies, this morphological trait could prioritize manoeuvrability and instantaneous thrust generation at the expense of span efficiency. Analogous contrasts are evident in Lepidoptera. The Monarch butterfly (*Danaus plexippus*), known for long-distance migration, possesses a high-efficiency (Ortega Ancel et al. 2017), quasi-elliptical planform. In contrast, species such as the Oakleaf butterfly (*Kallima inachus*) exhibit wing shapes that deviate significantly from the elliptical ideal to achieve crypsis (mimicry) (Wang et al. 2022). Our theoretical model estimates substantially lower span efficiency for the latter (Fig. 8), which can be interpreted as the aerodynamic cost incurred to enhance survival through mimicry that is now measurable in joules. Notably, our simplified theoretical model successfully captured these efficiency disparities driven by ecological contexts at the LM level with the interspecies differences in span efficiency being 3% and 30% with gliding and rotary wing analysis, respectively. This demonstrates the utility of our approach as a tool for evaluating baseline flight performance solely from wing morphology without the need for complex experimental setups. Furthermore, this method holds promise for inferring the flight capabilities of extinct species, where often only fossilized morphological data remains, allowing for estimation of flight performance of the fourth, extinct, group of active animal flyers—the pterosaurs.

## Conclusion

In this study, we integrated span efficiency data from multiple species of insects, birds, and bats measured previously in wind tunnel experiments to conduct a meta-analysis. Prior to the analysis, CFD demonstrated that discrepancies in the wake integration range (tail length) across past experimental data were critical factor affecting span efficiency calculations. Based on these findings, we standardized the entire dataset prior to phylogenetic comparative analyses that revealed that span efficiency strongly correlates with body size (mass). This suggests that larger flying animals may be under greater pressure to achieve high span efficiency. Specifically, we infer that this size-dependent sensitivity to induced drag costs acts as physical driver, forcing larger species to converge toward this higher efficiency. Simultaneously, theoretical span efficiencies for gliding and rotating flight exhibited significant correlations with experimental values. These results shed new light on how biological flight is shaped by the interplay between universal physical constraints, arising from scaling and the aerodynamic properties of wing morphology, and lineage-specific adaptive strategies, such as kinematics and wing structure within each clade. To show the utility of this morphology-based theoretical prediction, we compared the oakleaf butterfly with the famously migratory monarch butterfly. The monarch exhibits a significantly higher predicted span efficiency, illustrating the energetic trade-off between aerodynamic efficiency and crypsis. Our approach provides a versatile framework for evaluating baseline flight performance directly from the wing morphology of target organisms without requiring expensive apparatus and wind tunnel experiments, thereby contributing to future research in evolutionary biology and biomechanics.

## Supplementary Material

icag077_Supplemental_Files

## Data Availability

All data are incorporated into the article and its online supplementary material.

## Notes

From the symposium “Dinosaurs to Drones: Comparative Approaches to Understanding Flight.” presented at the annual meeting of the Society for Integrative and Comparative Biology, January 3–7, 2026.
